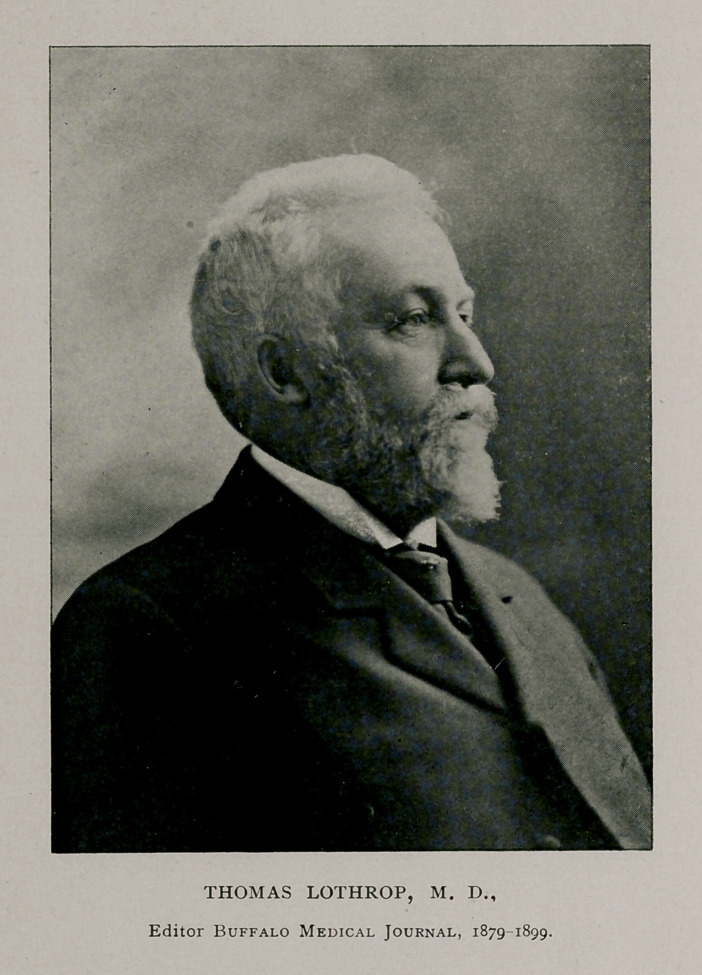# Dr. Lothrop.—Volume LV

**Published:** 1899-08

**Authors:** 


*EDITOR:*

WILLIAM WARREN POTTER, M. D.

All communications, whether of a literary or business nature and books for review, should be addressed to the editor: 284 FRANKLIN STREET, BUFFALO, N. Y.

VOL. XXXIX.—LV. AUGUST, 1899. NO. 1.

### DR. LOTHROP.—VOLUME LV.

DR. THOMAS LOTHROP, whose name has stood at the head of the editorial department of this Journal for twenty years, has severed his relations with the magazine and has transferred his interest to Dr. William Warren Potter, who thereby becomes sole owner and editor-in-chief. It is proper that the Journal should make comment appropriate to an event so significant in its history.

Very few men have sustained editorial relations to a medical journal for so long a period as twenty years and this circumstance, alone, speaks strongly of the character and individuality of the distinguished retiring editor. During a considerable part of the time he maintained relationship to the Journal Dr. Lothrop was an active, if not a leading spirit in the conduct of its affairs. His ready and forceful pen was often wielded in its columns and his tactful business acumen guided its career on the road to success. He has occupied a position of prominence in the medical profession during all this time and today stands second to none in all that constitutes a strong physician, a useful citizen and an upright man.

It is with a view to lessen his responsibilities that Dr. Lothrop takes leave of the Journal and it is needless to add that the Journal parts from him with regret. It hopes, however, to have the pleasure of printing an occasional contribution from his pen, in which case our readers will not lose the benefit of his professional experience. He has a fund of valuable material that could be utilised and he will do himself sad justice, as well as subject his colleagues to great disappointment, if he fails to use it for the benefit and advancement of medicine. He is yet in the prime of life and in the possession of a physical vigor that gives promise of continued usefulness for many years, as the portrait we publish from a recent photograph bears testimony.

We feel sure that every reader of the Journal who knows Dr. Lothrop will join with us in the sentiments we have expressed, as well as in tendering to him best wishes for a long continuance of his prosperous and useful life.

The Journal enters upon the fifty-fifth year of its publication with two additions to its editorial staff. Dr. Nelson W. Wilson, who has had a wide journalistic experience in New York and Buffalo, becomes assistant editor. Dr. Wilson's contributions to the Journal have been well received and he enters upon his duties with enthusiasm. Dr. Maud J. Frye, one of the best known of Buffalo's medical women, has earned her place as an associate editor through a high class of editorial work heretofore performed. It is needless for us to add that the Journal is more than pleased to acknowledge the faithful and meritorious services of Dr. William C. Krauss, who becomes an assistant editor. The other members of our editorial staff, Drs. James Wright Putnam, John Parmenter, Ernest Wende, John A. Miller and A. A. Hubbell, are entitled to our grateful thanks for valuable services and loyal interest in the welfare of the magazine. Our readers may be sure of a continued determination on the part of the entire staff to make the Journal grow better and better with each succeeding year.

We invite especially the support of the medical profession in Western New York to the end that the Journal may attain the ideal that it has always aimed at—namely, to be a creditable exponent of the science and art of medicine for the region it represents.

William Warren Potter

## Figures and Tables

**Figure f1:**